# Iconicity in Signed and Spoken Vocabulary: A Comparison Between American Sign Language, British Sign Language, English, and Spanish

**DOI:** 10.3389/fpsyg.2018.01433

**Published:** 2018-08-14

**Authors:** Marcus Perlman, Hannah Little, Bill Thompson, Robin L. Thompson

**Affiliations:** ^1^Department of English Language and Applied Linguistic, University of Birmingham, Birmingham, United Kingdom; ^2^Department of Applied Sciences, University of the West of England, Bristol, United Kingdom; ^3^Language and Cognition Department, Max Planck Institute of Psycholinguistics, Nijmegen, Netherlands; ^4^School of Psychology, University of Birmingham, Birmingham, United Kingdom

**Keywords:** sign language, spoken language, iconicity, modality, American Sign Language, British Sign Language, English, Spanish

## Abstract

Considerable evidence now shows that all languages, signed and spoken, exhibit a significant amount of iconicity. We examined how the visual-gestural modality of signed languages facilitates iconicity for different kinds of lexical meanings compared to the auditory-vocal modality of spoken languages. We used iconicity ratings of hundreds of signs and words to compare iconicity across the vocabularies of two signed languages – American Sign Language and British Sign Language, and two spoken languages – English and Spanish. We examined (1) the correlation in iconicity ratings between the languages; (2) the relationship between iconicity and an array of semantic variables (ratings of concreteness, sensory experience, imageability, perceptual strength of vision, audition, touch, smell and taste); (3) how iconicity varies between broad lexical classes (nouns, verbs, adjectives, grammatical words and adverbs); and (4) between more specific semantic categories (e.g., manual actions, clothes, colors). The results show several notable patterns that characterize how iconicity is spread across the four vocabularies. There were significant correlations in the iconicity ratings between the four languages, including English with ASL, BSL, and Spanish. The highest correlation was between ASL and BSL, suggesting iconicity may be more transparent in signs than words. In each language, iconicity was distributed according to the semantic variables in ways that reflect the semiotic affordances of the modality (e.g., more concrete meanings more iconic in signs, not words; more auditory meanings more iconic in words, not signs; more tactile meanings more iconic in both signs and words). Analysis of the 220 meanings with ratings in all four languages further showed characteristic patterns of iconicity across broad and specific semantic domains, including those that distinguished between signed and spoken languages (e.g., verbs more iconic in ASL, BSL, and English, but not Spanish; manual actions especially iconic in ASL and BSL; adjectives more iconic in English and Spanish; color words especially low in iconicity in ASL and BSL). These findings provide the first quantitative account of how iconicity is spread across the lexicons of signed languages in comparison to spoken languages.

## Introduction

Increasingly, language scientists recognize that iconicity – in complement to arbitrariness – is a fundamental feature of human languages (Perniss et al., 2010). On this theory, many of the forms of languages, from phonology to morphology to syntax, are motivated by a resemblance to the meaning they are used to express. Recent studies indicate that iconicity can serve several important functions, playing a role in how language is produced and processed, how it is learned and acquired by children, how it changes over history, and indeed, how it evolved in the first place (see reviews in Imai and Kita, 2014; Perlman and Cain, 2014; Perniss and Vigliocco, 2014; Dingemanse et al., 2015; Meir and Tkachman, 2018).

Building on the growing documentation of iconic phenomena across languages both signed and spoken, including the spontaneous gestures that are integrated with sign and speech, researchers are beginning to apply a comparative perspective to the study of iconicity (e.g., Voeltz and Kilian-Hatz, 2001; Kita and Özyürek, 2003; Dingemanse, 2012; Padden et al., 2013; Perry et al., 2015; Östling et al., 2018). Although most previous research has focused separately on signed or spoken languages, a comparative approach raises fundamental questions related to the modality of language. How is iconicity manifested in languages that are signed, compared to those that are spoken? Is it true that signed languages are categorically more iconic than spoken languages, as it is often assumed? Or might there be more interesting, richer differences – as well as similarities – in the patterns of iconicity found in signed and spoken languages?

In this paper, we examine whether the visual-gestural modality of signed languages facilitates iconicity for different kinds of lexical meanings than the auditory-vocal modality of spoken languages. Our study analyzes iconicity in the vocabularies of two signed languages – American Sign Language (ASL) and British Sign Language (BSL), and two spoken languages – English and Spanish. Using previously collected iconicity ratings of signs and words^1^, we directly compared how semantics motivates iconicity across the lexicons of the four different languages.

### Iconicity in Signed and Spoken Languages

It is widely taken for granted that signed languages are categorically more iconic than spoken languages. Many scholars have observed that signed languages, which are based on visible actions of the hands, body and face, are well suited for iconic representation (Johnston and Schembri, 1999; Meier, 2002; Meir, 2010; Cartmill et al., 2012; Kendon, 2014). Stemming from this potential, the iconicity in signed languages is widespread and clearly evident in both their grammar and, of focus here, their lexicon (Klima and Bellugi, 1979; Armstrong et al., 1995; Taub, 2001; Liddell, 2002; Aronoff et al., 2005). For example, Stokoe (1965) identified 25% of ASL signs to be either pantomimic or iconic, and Wescott (1971) further estimated that of the remaining 75% of signs, about two-thirds seemed plausibly derived from iconic origins. A more rigorous analysis examining 1944 signs of Italian Sign Language found that 50% of handshapes and 67% of body locations appeared to have iconic motivation (Pietrandrea, 2002). Indeed, many signs are iconic to such a degree that it was not until the pioneering work of Stokoe (1965) that they were even recognized by linguists as the components of legitimate languages, rather than idiosyncratic pantomimes and “mimic” signs (Wilcox, 2004).

Recent studies show that iconicity in the vocabularies of signed languages is not exhibited haphazardly. Signs for some kinds of meanings tend to be more iconic than others. For example, an analysis of BSL found that signs for objects and actions were more iconic than signs for properties (Perniss et al., 2017), presumably because manual signals afford more iconicity for objects and actions. Some patterns of iconicity have been shown to be common across a large number of signed languages. Lepic et al. (2016) found, across four unrelated languages, that two-handed signs are more frequently associated with meanings that are inherently “plural.” A larger-scale study based on the automated visual processing of signs from 31 different languages similarly found a correlation between the use of two-handed forms for signs and the degree of plurality in their meaning (Östling et al., 2018). Östling et al. (2018) also analyzed signs with sensory and body part-related meanings, where they found a correlation between the anatomical meaning of a sign and the location on the signer’s body where it is articulated.

In addition, comparative studies – such as by Meir et al. (2013) – have observed that patterns of iconicity in the lexicon of a signed language can vary systematically between languages. For example, Padden et al. (2013) examined the iconic strategies that signers used to represent hand-held tools (e.g., comb, mop, handsaw) in ASL, New Zealand Sign Language (NZSL), and Al-Sayyid Bedouin Sign Language (ABSL). Their analysis compared the use of three primary iconic strategies used by signers to represent the objects: signing as if handling the object (handling); signing as if using the object, but with the hands shaped to display qualities of its shape (instrument); and signing as if the hands are the object, but without performing its characteristic action (object). The results showed that, compared to hearing non-signers, signers of all three languages more strongly preferred the instrument strategy over the handling strategy. Notably, the signers of different languages also showed different proclivities: signers of ASL and ABSL displayed a stronger preference for the instrument strategy than NZSL signers.

In another comparative study, Meir et al. (2013) examined how signers use their body as a resource for the iconic representation of actions involving particular body parts. The authors observed that signers can make use of two different iconic strategies with respect to indicating the participants of an action. They can use their body as the subject of an action (e.g., the signer represents the subject of ‘eat’, i.e., the eater), without indicating the person of the participant. Or they can use their body to indicate a first person participant in an action in opposition to directing the sign toward locations in space associated with non-first person participants (representing ‘I eat’ vs. ‘you eat’). Meir et al. (2013) then compared the strategies used in the signs of ABSL, a young signed language, to those of Israeli Sign Language (ISL), a more mature language. For ISL, signs were elicited from three different age groups of signers, providing diachronic perspective on the language. The study found that ABSL signers only used signs implementing the body-as-subject strategy, without encoding person distinctions – a pattern that was also predominant with older ISL signers. In contrast, younger ISL signers – representing the more mature stage of the language – made use of a person agreement strategy in which they directed some signs toward locations in space to show who did what to whom. Thus, the body-as-subject strategy appears to be more basic and prevalent across signed languages, whereas the agreement strategy within ISL may be adopted by more mature languages in which a lexical class of agreement verbs is created over time.

In contrast to signed languages, the predominant linguistic theories over history have widely assumed that the vocabularies of spoken languages are essentially arbitrary (e.g., de Saussure, 1959; Hockett, 1960; Pinker and Bloom, 1990). A common line of explanation for this builds on the assumption that the auditory-vocal modality affords far less potential for iconicity than the visual-gestural modality (e.g., Hockett, 1978; Armstrong and Wilcox, 2007; Tomasello, 2008; Meir et al., 2013). The clear exception of onomatopoeia – the iconic representation of sounds – has often been trivialized, without much attempt at rigorous empirical justification. For example, Newmeyer (1992), citing Whitney (1874), referred to “the almost complete non-existence of an iconic relationship between words and their referents,” suggesting that “the number of pictorial, imitative, or onomatopoeic non-derived words in any language is vanishingly small” (p. 758)^2^. Similarly, Pinker (1999) observed that “onomatopoeia and sound symbolism certainly exist, but they are asterisks to the far more important principle of the arbitrary sign” (p. 2). And in this vein, a popular introduction to psycholinguistics acknowledges that onomatopoeic words such as *cuckoo*, *pop*, *bang*, *slurp*, and *squish* are exceptions to the principle of arbitrariness, but observes that “there are relatively few of these in any language” (Aitchison, 2007, p. 29).

Nevertheless, over the years, researchers have collected wide-ranging evidence of iconicity in the vocabularies of spoken languages (Perniss et al., 2010; Perlman and Cain, 2014; Dingemanse et al., 2015). As a baseline, it turns out that onomatopoetic words are much more prevalent than the above quotes suggest, and indeed, may even constitute a distinct lexical class that is universal across languages (Dingemanse, 2012). For example, although English has been characterized as a spoken language with a vocabulary that is relatively lacking in iconicity (e.g., Vigliocco et al., 2014), studies of onomatopoeic words in English reveal a substantial and varied inventory. For instance, an analysis by Rhodes (1994) examined over one hundred English words used to refer to “aural images,” including predominantly onomatopoeic words. These words spanned diverse conceptual categories, including sounds produced by the vocal tracts of humans (e.g., *yell, hum)*, and other animals (e.g., *moo, tweet*), as well as non-vocal sounds (e.g., *click*, *bang*). While somewhat narrower in scope, the use of iconic words to represent vocal tract actions in spoken languages can be seen as an analog to the iconic representation of various kinds of manual actions in signed languages.

Rhodes (1994) noted that onomatopoeic words fall broadly along a continuum in the degree to which they are conventionalized into the lexicon (also see Dingemanse and Akita, 2017). On one end are tame words, which are highly lexicalized and characterized by standard phonological and syntactic patterns. On the farthest end of tame, Rhodes observed that a few aural images are conveyed by standard arbitrary words such as *noise, sound,* and *din*. On the other end of the scale are wild words, which utilize the full range of the vocal tract to precisely imitate sounds (also see Lemaitre et al., 2016). Both the prevalence and the productiveness of onomatopoeia in English are illustrated in dictionaries, such as the online *Written Sound Onomatopoeia Dictionary*, which contains 772 entries^3^ (retrieved 1/11/2017), and *KA-BOOM!* – a dictionary of onomatopoeia in comic books – which contains 119 pages and thousands of entries (Taylor, 2007). The quickly evolving contents of these dictionaries pay tribute to the dynamic quality of onomatopoeia, which can serve as a productive source of lexical innovation, perhaps comparable in ways to the creative functions of iconicity in signed languages (cf. Klima and Bellugi, 1979).

Beyond the often-underestimated base of onomatopoeia, a growing number of cross-linguistic studies show that iconicity in spoken languages is far from limited to the representation of sounds. In most languages around the world, onomatopoeic words typically represent just a portion of a semantically broader class of vocabulary – variously termed mimetics, expressives, phonaesthemes, and most generically, *ideophones* – that are used to communicate about an array of concepts related to the senses (Diffloth, 1972; Nuckolls, 1999; Voeltz and Kilian-Hatz, 2001; Dingemanse, 2012; Kwon, 2015). As a general class, ideophones are characterized as marked words that are used to convey sensory imagery (Dingemanse, 2012). They are noted for their special forms and distinct grammatical properties, e.g., the use of reduplication as an iconic representation of repetition. Ideophones subsume onomatopoeia, and they broadly comprise a dynamic class of words that is commonly associated with creativity and lexical innovation (Dingemanse, 2014). In addition to sound, ideophones are used to express meanings from varied semantic domains such as luminance, manner of movement, size, texture, shape, taste, temperature, and emotional and psychological states (Dingemanse, 2012). This range is illustrated by Dingemanse (2012, p. 661), which presented an assortment of examples from seven diverse languages: for example, *gùdùù* ‘pitch dark’ (Siwu), *juluq* ‘to gulp down (something solid) without chewing’ (Somali), *dzing* ‘a sudden awareness or intuition, especially one that causes fright’ (Pastaza Quechua), *potïl* ‘soft and tender (surface)’ (Korean), *kiláaaa-kiláaaa* ‘in a zigzagging motion’ (Ngbaka Gbaya), *liplip* ‘sparkling like a diamond or piece of glass’ (Upper Necaxa Totonac), and *blbbbbccc* ‘painful embarrassment’ (Semai).

Native speakers typically have the impression that ideophones are distinctly depictive and often iconic of their meaning, and comprehension experiments with naïve listeners provide some support for these intuitions (Kantartzis et al., 2011; Dingemanse et al., 2016; also see Kwon, 2017 for English phonaesthemes). For example, Dingemanse et al. (2016) tested the ability of naïve listeners to understand the meanings of ideophones from a diverse sample of unfamiliar languages. The stimuli represented five different semantic domains, including color/vision, motion, shape, sound, and texture. Although listeners were most accurate at guessing the meanings of ideophones for sound concepts, their guessing was significantly above chance for each domain.

In light of cross-linguistic surveys indicating the widespread prevalence of ideophones, and especially their semantic diversity, some linguists have proposed that studies of ideophones call for typological and comparative approaches (Diffloth, 1972; Voeltz and Kilian-Hatz, 2001; Dingemanse, 2012, 2017a). In this spirit, Dingemanse (2012, p. 663) proposed an implicational hierarchy for the semantic range of ideophone systems across languages. According to this hierarchy, almost all spoken languages have ideophones for sound concepts. As an ideophone system becomes richer and more varied, it tends to expand first to encompass meanings related to movement, then visual patterns and other sensory perceptions, and finally inner feelings and cognitive states. The prominence of sound in this semantic hierarchy may correspond to the higher potential for iconicity in sound-related ideophones (cf. Dingemanse et al., 2016).

In addition to ideophones (including onomatopoeia), a number of cross-linguistic studies show that some words outside of this distinct class may also be iconic, especially within a few particular semantic domains. For example, across many languages, words that express the concept of ‘small’ are more likely to have higher-frequency vowels, such as the high front vowel in the English *teeny*, compared to the low-frequency back vowel in *huge* (Ultan, 1978; Ohala, 1994; Haynie et al., 2014; Blasi et al., 2016). This may reflect the tendency for smaller things – particularly vocalizing animals – to emit higher-frequency sounds, compared to larger things, which tend to emit sounds of lower frequency. A similar pattern is reflected in male and female personal names, e.g., *Emily* and *Thomas* (Pitcher et al., 2013), and in indexical words used to refer to proximal and distal referents, e.g., *this* and *that*, *near* and *far* (Tanz, 1971; Ultan, 1978; Johansson and Zlatev, 2013).

Similar to signed languages in which the body serves as an iconic naming device for anatomical meanings (Meir et al., 2013), in spoken languages, we find an analog in the articulation of words used to refer to parts of the vocal tract (Wichmann et al., 2010; Urban, 2011; Blasi et al., 2016). For example, evidence from statistical studies across large, diverse samples of languages indicates that words for ‘lip’ tend to feature bilabial consonants (as do words for ‘breast,’ perhaps related to suckling). In addition, words for ‘nose’ tend to feature nasal phonemes, and words for ‘tongue’ the lateral /l/.

Considered together, these studies illustrate how iconicity is a prevalent characteristic of signed and spoken lexicons alike. Crucially, iconicity appears to be spread systematically across the semantic space of a language in ways that correspond with the iconic resources of its modality. For example, in signed languages, iconicity is high in words related to (non-vocal) bodily actions, whereas in spoken languages iconicity is more concentrated in words related to vocal tract actions and sounds. Indeed, Dingemanse et al. (2015) has postulated some basic commonalities and differences that might characterize iconicity in the lexicons of signed versus spoken languages based on the semiotic affordances of each modality. They suggested that meanings related to qualities like ‘size,’ ‘repetition,’ ‘temporal unfolding,’ and ‘intensity’ may readily lend themselves to iconicity in both modalities. Meanings related to ‘spatial relations’ and ‘visual shape’ may afford iconicity in signed languages, but not spoken ones, while ‘sound’ and ‘loudness’ may afford considerable iconicity in spoken words, but not signs. Semantic domains like ‘abstract concepts’ and ‘logical operators’ may be hard for both types of languages to represent with iconic forms.

### Iconicity Ratings of Signs and Words

While cross-linguistic studies provide suggestive evidence for hypotheses such as those of Dingemanse et al. (2015), a more decisive investigation requires broader, systematic analyses of how iconicity is spread across the lexicons of individual languages. To this end, some recent studies have used iconicity ratings collected for large numbers of signs (e.g., Vinson et al., 2008; Caselli et al., 2017) and words (e.g., Perry et al., 2015; Winter et al., 2017a). For example, an original study of BSL collected iconicity ratings for 300 signs sampled from various sources to include a range of iconic and non-iconic signs (Vinson et al., 2008). The signs were rated by deaf signers on a scale from 1 (not iconic) to 7 (most iconic). The results showed that the iconicity of signs was negatively correlated with the age at which they are typically acquired: signs learned earlier tended to be more iconic (also see Thompson et al., 2012 for similar findings with children). There was also a small positive correlation between iconicity and the familiarity of signs. Iconicity ratings of a larger, more widely representative sample of ASL signs – these rated by hearing non-signers – found that signs were skewed toward the arbitrary end of the 1-to-7 scale (Caselli et al., 2017). Iconicity ratings showed a weak negative correlation with frequency, and a positive correlation with neighborhood density – that is, more iconic signs tended to be similar in form to more signs. A follow-up study with a subset of the ASL signs found a similar relationship between iconicity and age of acquisition to that of BSL (Caselli and Pyers, 2017). Thus, the use of iconicity ratings revealed interesting patterns in the distribution of iconicity across the lexicons of these two, unrelated signed languages.

Recently, a series of studies applied a similar approach to study iconicity in the vocabularies of spoken languages. The first of these studies compared iconicity in roughly 600 words in English and in Spanish (Perry et al., 2015). Notably, English and Spanish are Indo-European languages, which – it has been claimed – are less iconic than most other spoken languages (Perniss et al., 2010). This idea is illustrated by Vigliocco et al. (2014, p. 2): “Indeed, if we look at the lexicon of English (or that of other Indo-European languages), the idea that the relationship between a given word and its referent is defined by an arbitrary connection alone seems entirely reasonable. For example, there is nothing in the sequence of sounds in the English word ‘house’ that indicates its meaning of ‘a building for human habitation’.” However, contrary to this line of reasoning, the results of Perry et al. (2015) demonstrate that the vocabularies of English and Spanish are iconic in measurable, theoretically interesting ways. For example, in both languages, as in BSL and ASL, the iconicity ratings of words were negatively correlated with their age of acquisition – even when excluding onomatopoeia (also see Massaro and Perlman, 2017; Perry et al., 2017). Thus, it appears that young English and Spanish speaking children are sensitive to the iconicity of words, and they pick up on the more iconic words first.

The ratings also gave an opportunity to examine whether iconicity in languages like English and Spanish – which lack rich ideophone systems – might nevertheless pattern according to certain semantic dimensions, such as those postulated by Dingemanse (2012) and Dingemanse et al. (2015). Indeed, when iconicity was compared between lexical classes, some noteworthy patterns emerged. In English, Perry et al. (2015) found that onomatopoeia and interjections were highest in iconicity, followed by adjectives and verbs, then nouns, and finally closed-class function words. This pattern roughly corresponds with the ordering of Dingemanse’s (2012) implicational hierarchy, which proposed that ideophones are most prevalent for the expression of sound concepts, followed by concepts related to motion, vision, and other sensory perceptions. Similarly, Imai and Kita (2014) noted that ideophones typically have a rich inventory for expressing manners of action, physical sensations and certain properties of objects, but are not often used to refer directly to objects. Thus, it fits that in English, onomatopoeia, and then verbs – typically relating to motion and action, and adjectives – relating to sensations and properties, would be most iconic. Likewise, it fits that nouns, which often refer to objects, would be relatively low in iconicity. Furthermore, the low ratings for function words may reflect Dingemanse et al.’s (2015) suggestion that logical relations are not amenable to iconic representations.

The results for Spanish were comparable to those of English, but with one key difference that may stem from a typological difference between the languages. Perry et al. (2015) noted that English and Spanish vary in the typology of their verbs (Talmy, 2000; Beavers et al., 2010). English is a satellite-framed language, which typically conveys manner of motion in the main verb. In contrast, Spanish is a verb-framed language – verbs tend to convey the path of motion, and leave information about the manner for expression in adverbials. For example, consider the English sentence “The bottle floated into the cave,” in which manner of movement is expressed by the verb. Compare this to the Spanish “La botella entró a la cueva flotando,” in which the manner of the action, “flotando” (floating) is separated from the main verb “entró” (moved-in). Because Spanish verbs tend not to express manner of motion, a rich source of iconicity in many ideophones, Perry et al. (2015) predicted that these words would be less iconic. In line with this hypothesis, the results showed that the iconicity of Spanish verbs was low compared to adjectives, and more comparable to nouns and function words.

A subsequent study with English expanded on Perry et al.’s (2015) iconicity ratings to include a total of 3001 words (Winter et al., 2017a). This study found essentially the same pattern of results with respect to lexical class: onomatopoeia and interjections were highest in iconicity, then verbs, adjectives, nouns, and finally function words. Winter et al. further examined the specific semantic factors that might influence the distribution of iconicity across English vocabulary. First, they tested whether the iconicity ratings correlated with ratings of the degree to which a word “evokes a sensory experience” (Juhasz and Yap, 2013). In a model that also included imageability ratings and frequency, sensory experience was the strongest predictor of iconicity. As with ASL (Caselli et al., 2017), frequency was negatively correlated with iconicity. Imageability was also negatively correlated with iconicity, suggesting that more highly visual words may be less iconic. A subsequent analysis categorized the meanings of the words into their dominant sensory modality. This showed that words with meanings most strongly associated with the auditory and the tactile modalities were rated higher in iconicity than those associated with the other modalities, with visual words even lower than olfactory and gustatory words.

Another set of analyses by Sidhu and Pexman (2017) with the English iconicity ratings found a similar relationship between iconicity and the sensory experience ratings of Juhasz and Yap (2013). Additionally, this study found that the strongest relationship was between iconicity and semantic neighborhood density, which mediated the effect of sensory experience. Words in sparser semantic neighborhoods, particularly those high in sensory experience, tended to be more iconic, a result that held across adjectives and adverbs, verbs, and nouns. This finding supports the idea that lexicons exhibit a balance between iconicity and arbitrariness: as more words share similar meanings, the ability to discriminate between them becomes more critical, which drives them toward more arbitrary forms (Gasser, 2004; Dingemanse et al., 2015).

In summary, these studies with iconicity ratings show some of the various ways that iconicity is systematically distributed across the lexicons of signed and spoken languages. Some of these patterns appear to be common to both language modalities. For example, there is a consistent relationship between the iconicity of a sign or word and the age at which it is learned by children: more iconic items tend to be learned earlier. Yet, these studies also hint at some notable differences between the iconicity in signed and spoken vocabularies, particularly with respect to different semantic domains. For instance, in spoken languages, adjectives and particularly words for auditory and tactile properties appear to be relatively high in iconicity, whereas this may not be the case in signed languages (cf. Perniss et al., 2017). Conversely, nouns and visual words in spoken languages appear to be low in iconicity, while there is reason to think they are more highly iconic in signed languages.

### Current Study

In the current study, we conducted a direct comparison between the iconicity of signed and spoken vocabularies and how it varies across different semantic domains. We asked whether the gestural-visual modality of signed languages motivates iconicity for different kinds of meanings than the vocal-auditory modality of spoken languages. To investigate this question, we utilized previously collected iconicity ratings to compare ASL and BSL with English and Spanish. Across the four languages, we examined: (1) the correlations between languages for iconicity ratings of the same meanings; (2) the relationship between iconicity ratings and an array of ratings for various semantic properties (e.g., concreteness, sensory experience); (3) how iconicity ratings vary broadly between (English-based) lexical classes; and (4) how they vary between more specific semantic categories (e.g., clothes, colors).

## Materials and Methods

### Iconicity Ratings

Our study utilized previously collected iconicity ratings for 100s of signs and words in ASL, BSL, English, and Spanish. These samples of signed and spoken languages were chosen opportunistically because of the pre-existing data available, and not because ASL and BSL share any particular comparable relationship to English and Spanish. Notably, ASL and BSL are not historically related to each other, whereas English and Spanish share common ancestry as Indo-European languages. Moreover, there is even more recent shared ancestry between the spoken languages as English contains a large amount of Latinate vocabulary from French.

**Table 1** shows information about the source of the ratings for each language, the participants who provided the ratings, and the number of signs and words covered. Iconicity ratings for 993 ASL signs come from Caselli et al. (2017; see the LEX-ASL database^4^). The signs were rated by non-signers on a scale from 1 (“not iconic at all”) to 7 (“extremely iconic”). Iconicity ratings for 604 BSL signs are from Vinson et al. (2008) and Thompson et al. (unpublished). In these studies, signs were rated 1 (arbitrary) to 7 (iconic) by a mix of native and non-signers in four different experiments. When ratings for a given BSL sign were collected in multiple experiments, we used an averaged rating for our analysis. We found that our different ratings for BSL signs were highly correlated, including between signers and non-signers (*r* ≥ 0.84 for all sets of overlapping ratings). This was comparable to a study of ASL with a different set of signs, which found a correlation of *r* = 0.82 between the ratings of ASL signs by signers and non-signers (Sevcikova Sehyr et al., 2017).

**Table 1:** Source of iconicity ratings for each language.

Modality	Language	No. signs	Raters	References
Signed	ASL	993	Non-signers	Caselli et al., 2017
	BSL	604	Mix of native/non-signers	Vinson et al., 2008; Thompson et al., unpublished
Spoken	English	3001	Native speakers	Perry et al., 2015; Winter et al., 2017a
	Spanish	637	Native speakers	Perry et al., 2015

Iconicity ratings for 3001 English words were collected by Perry et al. (2015) and Winter et al. (2017a). Native speakers rated the words on a scale from -5 (sounds like the opposite of what it means) to 5 (sounds like what it means), with 0 (arbitrary) at the middle point. Iconicity ratings for 637 Spanish words come from Perry et al. (2015). These were provided by native speakers and collected according to a similar procedure as the English ratings. In a few instances, multiple Spanish words shared the same English gloss, e.g., Spanish *un* and *una* translate to English *a*, and Spanish *poco* and *poquito* to English *little*. For these cases, we selected the variant with the higher iconicity rating for our analyses.

We direct the reader to the original sources for further information on the procedures used to collect the ratings, including the particular instructions and examples used to define ‘iconicity.’ One detail of the instructions that is worth noting here is that they all reflected the modality of the language: for signs, iconicity was defined as when a sign ‘looks’ like what it means, and for spoken words, as when a word ‘sounds’ like what it means.

### Ratings for Semantic Properties

**FIGURE 1:**
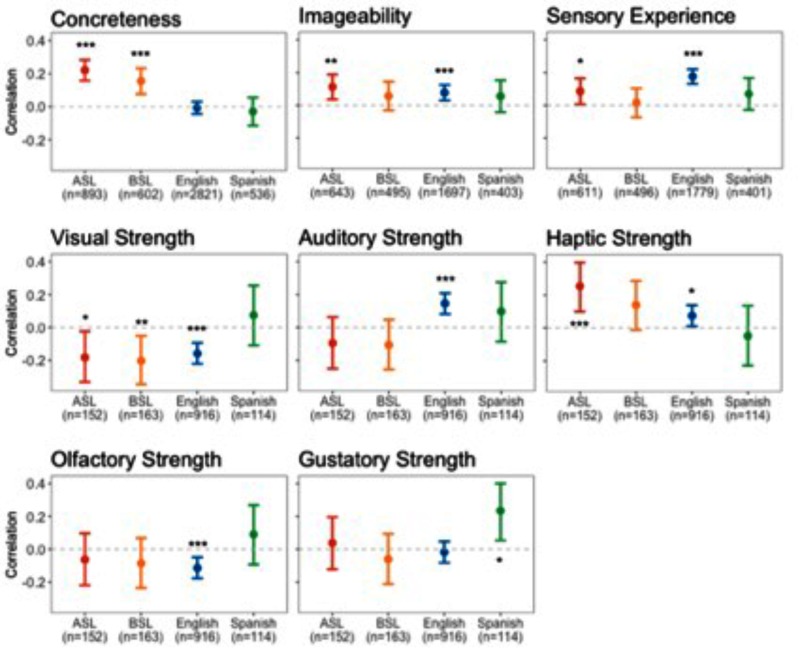
Pearson correlation coefficients between iconicity ratings and ratings of semantic properties. Error bars show 95% confidence interval. Below each language, *n* indicates the number of signs or words for which there were ratings. Stars indicate the significance of the correlation. ^∗∗∗^*p* < 0.001, ^∗∗^*p* < 0.01, *p*^∗^ < 0.05.

We investigated the relationship between the iconicity ratings and a battery of ratings related to various semantic properties of words: concreteness, imageability, sensory experience, and perceptual strength for vision, audition, touch, gustation, and olfaction. **Figure 1** shows the number of items in each language for which we had each measure. Notably, each of these measures was collected with respect to English words, and thus, our analyses of words and signs of other languages use the ratings from their English translations.

Concreteness ratings come from Brysbaert et al. (2014). Participants were asked to rate the degree to which the referent of a word was experienced “directly through one of the five senses.” Imageability ratings come from Cortese and Fugett (2004), which assessed how much the meanings of words related to “sensory experience, such as a mental picture of sound.” Sensory experience ratings were collected by Juhasz and Yap (2013). For these ratings, participants rated the degree to which a word evoked a sensory experience. Finally, perceptual strength ratings include ratings of verbs from Winter (2016), adjectives from Lynott and Connell (2009), and nouns from Lynott and Connell (2013). These ratings measured the degree to which a word was associated with each of the five sensory modalities.

### Lexical Classes

Our analyses of iconicity and lexical class focused on the 220 meanings for which we had iconicity ratings in all four languages. To sort the meanings into broad semantic categories, we assigned each one to a lexical class based on its part of speech in English, adapted from Brysbaert and Keuleers’ (2012) annotation of the SUBTLEX corpus. This resulted in 132 nouns, 41 verbs, 28 adjectives, and 19 grammatical words and adverbs (not typically related to manner). **Table 2** shows the lexical class for each of these words.

**Table 2:** Lexical class and particular semantic categories of the 220 meanings for which we had iconicity ratings in all four languages.

Lexical class	Semantic category	Meanings
Nouns	Small artifacts (roughly fits within two hands)	Ball, balloon, book, bottle, doll, hammer, knife, medicine, money, napkin, paper, pencil, scissors, soap, spoon, telephone
	Body parts and clothes	Arm, dress, ear, glasses, hair, hat, mouth, necklace, scarf, shirt, shorts, shoulder, skirt
	Vehicles	Airplane, bicycle, boat, car, helicopter, motorcycle, train
	Food	Apple, banana, bread, butter, cake, cereal, cheese, chocolate, coffee, egg, hamburger, meat, milk, potato, salt, soup, spaghetti, strawberry
	Animals	Animal, bear, bird, butterfly, cat, cow, crocodile, deer, dog, duck, elephant, giraffe, horse, lion, monkey, mouse, owl, pig, rabbit, sheep, snake, squirrel, tiger, turkey, turtle, wolf
	Natural things	Cloud, fire, flower, grass, moon, rain, star, sun, tree, water
	Buildings and rooms	Bedroom, church, garage, house, kitchen, school
	People	Baby, boy, brother, clown, doctor, family, friend, girl, man, nurse, person, sister, uncle, woman
	Other things (bigger and/or more abstract things)	Bed, blanket, box, chair, clock, computer, country, day, door, dryer, flag, mirror, morning, movie, night, picture, pillow, shower, stairs, street, table, window
Adjectives	Colors	Black, blue, green, orange, red, yellow
	Evaluations	Bad, cute, good, poor, right
	Feelings and emotions	Cold, hungry, sad, scared, sick, tired
	Other properties	Dirty, full, hard, new, old, quiet, same, slow, small, tall, wet
Verbs	Manual actions	Brush, draw, drink, eat, paint, pull, push, sweep, throw, tie, write
	Locomotion	Come, fall, fly, go, jump, run, skate, swing, walk
	Other actions	Break, buy, cook, cry, find, finish, have, help, make, play, read, see, show, sing, sit, sleep, talk, think, wait, want, win
Adverbs and grammatical words	Spatial and determiners	Behind, here, more, some, that
	Time	After, later, now, yesterday
	Question words	How, what, when, where, which, who
	Other grammatical words	And, but, you, with

The use of lexical class to classify meanings into broad semantic categories is supported by theories of cognitive grammar, which posit that classes such as nouns, verbs, and adjectives reflect conceptual prototypes (Givón, 2001; Langacker, 2008; also see Strik Lievers and Winter, 2018). For example, according to Langacker, nouns are rooted in the prototype of a physical object, a ‘thing’ (i.e., subsuming people and places and not limited to physical entities); verbs typically refer to actions and events, profiling change over time; and adjectives typically specify more static properties. Nevertheless, we emphasize that our designated lexical categories, based on English, serve just for a broad-stroke comparison of how different kinds of meanings vary in iconicity between the languages. We do not mean to imply that ASL, BSL, and Spanish necessarily share these same lexical classes with English.

### Specific Semantic Categories

We further classified each of the 220 meanings into more specific semantic categories. As shown in **Table 2**, these included nine classes of nouns, three classes of verbs, four classes of adjectives, and four classes of grammatical words and adverbs. These specific categories were determined *ad hoc* from the sample of 220 meanings that happened to feature ratings in each language. Accordingly, their purpose is to present a more detailed – but exploratory – breakdown of iconicity across the four vocabularies.

### Data Availability

Data and analysis scripts are made available through the Open Science framework at https://osf.io/d759h/.

## Results and Discussion

### Correlation of Iconicity Between Languages

First, we calculated the correlation between the iconicity ratings of each pair of languages. The correlation between ASL and BSL signs was fairly strong, *r* = 0.68, *t*(344) = 17.0, *p* < 0.0001. English showed a small but reliable correlation with ASL, *r* = 0.16, *t*(550) = 3.7, *p* < 0.0001, BSL, *r* = 0.22, *t*(601) = 5.4, *p* = 0.0003, and Spanish, *r* = 0.16, *t*(478) = 3.6, *p* = 0.0003. Spanish ratings did not significantly correlate with BSL, *t*(323) = 0.2, *p* = 0.83, and showed a weak, negative correlation with ASL, *r* = -0.12, *t*(275) = -2.02, *p* = 0.04.

These results suggest that signs for particular meanings are fairly consistent in their level of iconicity in ASL and BSL, while there is greater variability between English and Spanish words. This pattern may reflect that potential iconic mappings between form and meaning are more direct and transparent for many signs, and hence more consistently realized across different signed languages. In comparison, words may reflect vaguer, less obvious iconic mappings between form and meaning, which, as a consequence, appear less consistently across spoken languages.

Intriguingly, in addition to being correlated with Spanish, the iconicity of English words was also weakly, but positively, correlated with the iconicity of the corresponding ASL and BSL signs. However, this was not the case with Spanish, which showed – if anything – a negative correlation with the signed languages. In part, this may stem from the low iconicity of verbs in Spanish in comparison to English, as was previously reported by Perry et al. (2015). Accordingly, English may share with ASL and BSL relatively high iconicity in verbs, but shares other features of iconic vocabulary with Spanish. The following analyses examine how iconicity is spread across these four vocabularies in more detail, shedding light on their commonalities and differences.

### Iconicity and Semantic Properties

For each language, we examined the relationship between iconicity ratings and ratings of a host of semantic properties: concreteness, imageability, sensory experience, and perceptual strength with respect to vision, audition, touch, gustation, and olfaction. **Figure 1** shows plots of the correlations between the iconicity ratings in each language and these variables. To test whether the strength of these relationships differed between language modalities (i.e., signed or spoken), we constructed linear mixed-effects models with the ratings of each semantic property as a predictor of iconicity ratings. The models included main effects for the semantic variable and modality (both centered), and a term for their interaction. Random intercepts were included for language and meaning, and random slopes were included for the semantic variable on language. Significance tests were calculated using χ^2^-tests that compared the model likelihoods with and without the factor of interest.

The model for concreteness ratings showed that concreteness was a significant predictor of iconicity, *b* = 0.14, 95% CI = [0.08, 0.20], χ_1_^2^= 4.42, *p* < 0.01. More concrete meanings tended to have more iconic signs and words. There was also a significant interaction between concreteness and modality, *b* = -0.28, 95% CI = [-0.40, -0.17], χ_1_^2^= 9.04, *p* < 0.01. This indicated that concreteness was more highly correlated with iconicity ratings in signed languages.

The model for sensory experience ratings showed that sensory experience was a significant predictor of iconicity ratings, *b* = 0.13, 95% CI = [0.05, 0.21], χ_1_^2^= 7.17, *p* < 0.01. Meanings higher in sensory experience were associated with more iconic signs and words. There was not a significant interaction between modality and sensory experience, χ_1_^2^= 0.96, n.s.

The model for imageability ratings (scaled to *z*-scores) showed that imageability was a significant predictor of iconicity ratings, *b* = 0.11, 95% CI = [0.04, 0.17], χ_1_^2^= 6.14, *p* < 0.05. More imageable meanings tended to have more iconic signs and words. There was not a significant interaction between imageability and modality, χ_1_^2^= 1.95, n.s.

The model for visual strength ratings showed that visual strength was a significant predictor of iconicity ratings, *b* = -0.18, 95% CI = [-0.29, -0.06], χ_1_^2^= 6.92, *p* < 0.01. Meanings with greater visual strength tended to have less iconic signs and words. There was also a significant interaction between visual strength and modality, *b* = 0.20, 95% CI = [0.01, 0.41], χ_1_^2^= 4.37, *p* < 0.05. The relationship between visual strength and iconicity ratings was more strongly negative in signed languages.

The model for auditory strength ratings showed that auditory strength was not a significant predictor of iconicity ratings, χ_1_^2^= 0.03, n.s. However, there was a significant interaction between auditory strength and modality, *b* = 0.21, 95% CI = [0.10, 0.33], χ_1_^2^= 7.52, *p* < 0.01. This revealed that the positive relationship between auditory strength and iconicity ratings was stronger in spoken languages.

The model for haptic strength ratings showed that haptic strength was a significant predictor of iconicity ratings, *b* = 0.14, 95% CI = [0.03, 0.25], χ_1_^2^= 5.10, *p* < 0.05. Meanings with greater haptic strength were associated with more iconic signs and words. There was a marginally significant interaction between haptic strength and modality, *b* = -0.21, 95% CI = [-0.42, 0.00], χ_1_^2^= 3.82, *p* = 0.05, suggesting that the relationship between haptic strength and iconicity was stronger in signed languages.

The model for gustatory strength ratings showed that gustatory strength was not a significant predictor of iconicity ratings, χ_1_^2^= 0.24, n.s., and there was no interaction with modality, χ_1_^2^= 0.14, n.s.

Finally, the model for olfactory strength ratings showed that olfactory strength was a significant predictor of iconicity ratings, *b* = -0.10, 95% CI = [-0.19, -0.02], χ_1_^2^= 3.14, *p* < 0.05. Across languages, the olfactory strength of meanings was negatively associated with iconicity ratings. There was not an interaction between olfactory strength and modality, χ_1_^2^= 0.18, n.s.

These results reveal several interesting patterns across the four languages in the relationship between iconicity and the semantics of signs and words. One notable finding is that iconicity is strongly associated with the concreteness of meanings in the signed languages, but not in the spoken languages. In comparison, while the correlation between iconicity with both sensory experience and imageability is weaker, it is found across the four languages. The relationship between sensory experience and iconicity in English matches previous results using much of the same data (Sidhu and Pexman, 2017; Winter et al., 2017a), although Winter et al. (2017a) found the opposite – a negative – relationship between iconicity and imageability. In this latter model, Winter et al. included additional factors, including sensory experience rating, which may have accounted for some of the variance explained by imageability in the present model.

A somewhat counterintuitive result was that ratings of visual perceptual strength were negatively correlated with iconicity ratings in both signed languages. Part of the explanation for this may stem from meanings referring to color (e.g., *red*, *blue*, *black*), which are among the meanings with the strongest visual strength. To examine this possibility, we removed color words from the set, and then retested the model of visual strength ratings as a predictor of iconicity ratings. This showed a reduced, but still significant negative effect of visual strength *b* = -0.13, 95% CI = [-0.24, -0.02], χ_1_^2^= 5.38, *p* < 0.05). However, the interaction between visual strength and modality was no longer significant, χ_1_^2^= 0.89, *p* = n.s. Thus, while visual strength was still negatively correlated with iconicity ratings across the languages, after removing color words, this relationship was weaker overall, particularly within the signed languages.

Along with concreteness, haptic strength proved to be the strongest positive predictor of iconicity ratings, both overall, and especially in signed languages. For signed languages, this is an intuitive finding. The haptic sense is largely channeled through manipulative actions of the hands, and therefore, these meanings may afford a high degree of iconicity in signs. The positive correlation between haptic strength and iconicity in English fits with the similar finding by Winter et al. (2017a), which used mostly the same data. Ongoing work suggests that part of the basis for the high iconicity of tactile words may relate to surface texture, and particularly the dimension of roughness versus smoothness (Winter et al., 2017b). However, Spanish appears to contradict this trend common to English and the two signed languages.

As expected, auditory strength was a strong predictor of iconicity in the spoken languages in particular, with an opposing tendency in ASL and BSL. This again replicates Winter et al. (2017a) for English. These results likely reflect the highly compatible format of the vocal-auditory modality of speech for the iconic representation of sound-related meanings (e.g., Perlman and Cain, 2014; Dingemanse et al., 2015).

Across the four languages, the relationship between gustatory and olfactory strength and iconicity was less consistent. For the signed languages and English, it appears to be, if anything, a somewhat negative relationship. Meanings strongly associated with smell and taste tended to have less iconic forms. Spanish, on the other hand, hints at the opposite: a positive relationship between iconicity and meanings related to smell and taste. These preliminary – and tentative – findings with Spanish are unexpected. Smell and taste are distinct from the sensory modalities primarily involved in signed and spoken communication, which directly involve vision, audition and the kinesthetic sense, vis-a-vis the visual and auditory perception of the sights and sounds of bodily movements. And while meanings related to smell and taste are represented by ideophones across languages, they have been counted as less common (Dingemanse, 2012).

In interpreting these different results, it should be considered that all of the ratings for semantic properties were based on the ratings of English glosses judged by English speakers. Thus, the way these ratings characterize the semantics of the translated ASL and BSL signs and the Spanish words is likely to be inaccurate to a degree. Additionally, as a result of this procedure, more English words were covered by the ratings than were the signs and words of the other languages. Consequently, our inferences about English may be more finely tuned than those for the other languages. Conversely, the fewest items were covered for Spanish, leading to wider margins for error in our estimates.

### Iconicity and Lexical Classes

**FIGURE 2:**
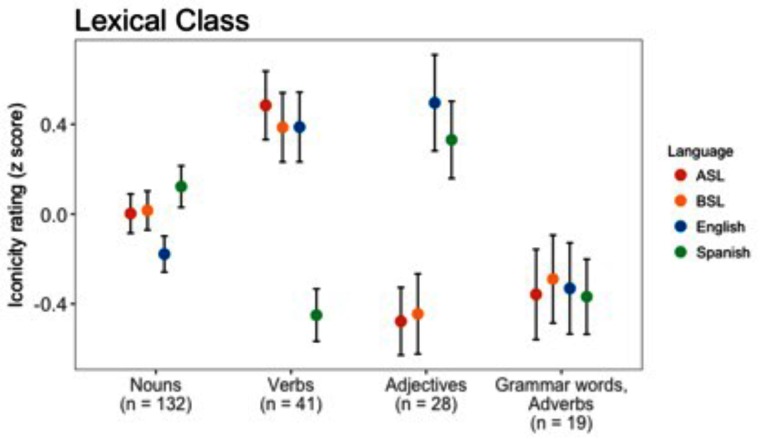
Means and standard errors of normalized iconicity ratings for each language by English lexical class. The values were calculated from the 220 meanings for which we had iconicity ratings in all four languages. n indicates the number of shared words and signs for each lexical class.

In the next set of analyses, we focused on the 220 meanings for which we had iconicity ratings in all four languages. First, we examined how iconicity varied across the vocabularies of the four languages according to broad semantic categories based on the lexical class of the English gloss. **Figure 2** shows iconicity ratings by lexical class – nouns, verbs, adjectives, and adverbs and grammatical words – for each language, displayed as *z*-scores. To test for differences in iconicity between lexical classes, for each language, we constructed a generalized linear model with lexical class as a predictor of iconicity rating.

For ASL, the model showed that nouns were less iconic than verbs, *b* = 0.86, 95% CI = [0.25, 1.48], *t* = 2.78, *p* < 0.01, but more iconic than adjectives, *b* = -0.86, 95% CI = [-1.57, -0.15], *t* = -2.37, *p* < 0.05. Nouns were not significantly higher in iconicity than grammatical words (and adverbs), *b* = -0.65, 95% CI = [-1.48, 0.19], n.s. Similarly, for BSL, nouns were also lower in iconicity than verbs, *b* = 0.62, 95% CI = [0.04, 1.21], *t* = 2.10, *p* < 0.05, but higher than adjectives, *b* = -0.78, 95% CI = [-1.45, -0.10], *t* = -2.24, *p* < 0.05. Again, nouns were not significantly more iconic than grammatical words, *b* = -0.51, 95% CI = [-1.31, 0.28], n.s. For English, nouns were lower in iconicity than both verbs, *b* = 0.49, 95% CI = [0.20, 0.78], *t* = 3.27, *p* < 0.01, and adjectives, *b =* 0.58, 95% CI = [0.24, 0.93], *t* = 3.46, *p* < 0.001, but there was no significant difference between nouns and grammatical words, *b* = -0.13, 95% CI = [-0.53, 0.27], n.s. And finally, the model for Spanish indicated no statistical difference in iconicity between nouns and adjectives, *b* = 0.17, 95% CI = [-0.15, 0.48], n.s., but nouns (and adjectives) were higher in iconicity than verbs, *b* = -0.46, 95% CI = [-0.73, -0.19], *t* = -3.24, *p* = 0.001. Nouns were also significantly higher in iconicity than grammatical words, *b* = -0.39, 95% CI = [-0.77, -0.02], *t* = -2.06, *p* < 0.05.

To determine whether there was an interaction between modality and lexical class, we constructed a linear mixed-effects model of iconicity rating. The model included main effects for (centered) modality and lexical class and a term for their interaction. Random intercepts were included for language and meaning. The results showed a main effect for lexical class, χ_1_^2^= 14.75, *p* < 0.01, indicating that overall, nouns were less iconic than verbs, *b* = 0.38, 95% CI = [0.08, 0.68], *t* = 2.52, and more iconic than grammatical words, *b* = -0.42, 95% CI = [-0.83, -0.02], *t* = -2.04, but not more iconic than adjectives, *b* = -0.22, 95% CI = [-0.57, 0.12], *t* = -1.26. There was a highly significant interaction between modality and lexical class, χ_1_^2^= 43.97, *p* < 0.001. This interaction reflected that adjectives were relatively higher in iconicity in spoken languages, *b* = 1.19, 95% CI = [0.48, 1.68], *t* = 4.86, and that verbs were higher in signed languages, *b* = -0.73, 95% CI = [-1.14, -0.31], *t* = -3.45. There was no evidence that the iconicity of grammatical words differed between modalities, *b* = 0.32, 95% CI = [-0.25, 0.87], *t* = 1.10.

These analyses point to some interesting differences between signed and spoken languages in how iconicity is spread across broad semantic categories of signs and words. In signed languages, verbs – and thus, presumably, actions – were consistently high in iconicity. This may derive from the natural correspondence between sign and action, as signs are themselves comprised of manual and bodily actions (Armstrong and Wilcox, 2007). Like Perry et al. (2015), we found that English verbs were also high in iconicity, while Spanish verbs were markedly lower. English verbs may be more iconic because they tend to express information about the manner of motion, in contrast to Spanish verbs which do not. Manner of motion might be especially amenable to iconic expression in speech, as, for example, reflected in ideophones (Imai and Kita, 2014).

Notably, in all four languages, nouns – which typically refer to various kinds of things – exhibited an average level of iconicity. Previous work in BSL has suggested that signs for objects, along with actions, are more likely to be iconic (Perniss et al., 2017). Yet, the current results suggest that signs for actions, on the whole, tend to be more iconic than signs for things. At least part of the explanation for this discrepancy may be that there are considerably more nouns in our analyses than other lexical classes. Thus, the nouns may extend to more abstract and complex meanings that are less well suited to iconicity.

As in previous studies (Perry et al., 2015; Winter et al., 2017a), adjectives, which contain many meanings for properties, are rated high in iconicity in both English and Spanish. This contrasts to ASL and BSL, in which adjectives were relatively low in iconicity, at least as compared to nouns and especially to verbs. Similarly, Perniss et al. (2017) found that BSL signs for properties tended not to be iconic. Such findings may be seen to fall out of line with accounts such as Dingemanse et al. (2015), proposing that meanings related to certain properties – such as ‘size,’ ‘repetition,’ ‘temporal unfolding,’ and ‘intensity’ – may lend themselves to iconicity in both modalities. One possible reason is that the iconicity for the apparently low degree of iconicity in signs for properties is that the iconicity of signed vocabularies is dominated by even more easily representable actions, and to a lesser degree, things. Or it may be that many properties (e.g., colors) do not, in fact, readily lend themselves to iconicity through the manual movements of signs.

Finally, we observed that the miscellaneous category of grammatical words and adverbs tended to be relatively low in iconicity across both the signed and spoken languages. This conclusion is limited by the smaller sample of these meanings, but replicates previous results in English from Perry et al. (2015) and Winter et al. (2017a) with much of the same data. It fits with the prediction of Dingemanse et al. (2015) that meanings like ‘abstract concepts’ and ‘logical operators’ may be hard for both types of languages to represent with iconic forms.

### Iconicity and Specific Semantic Categories

**FIGURE 3:**
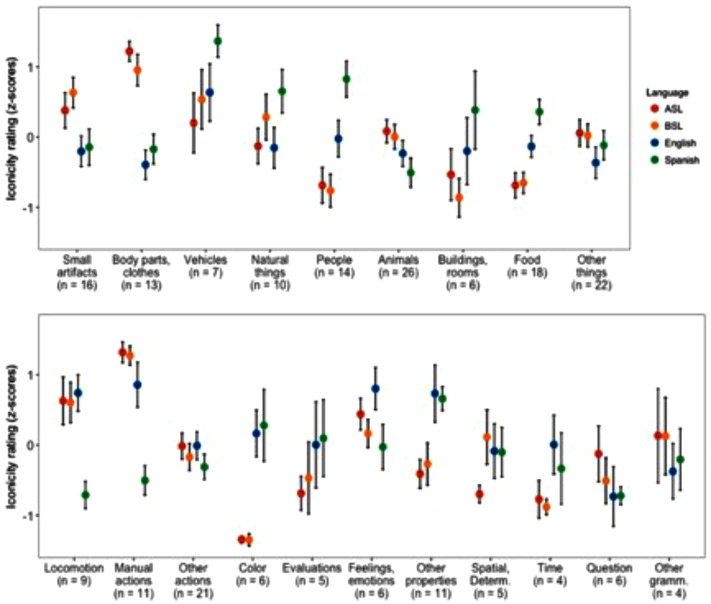
Means and standard errors of normalized iconicity ratings by specific semantic category. **(Top)** Shows categories of nouns. **(Bottom)** Shows categories spanning adjectives, verbs, and other lexical classes.

Finally, we zoomed in and looked at iconicity across more specific semantic categories for the same 220 meanings. The top panel of **Figure 3** shows the means and standard errors of the *z*-scored iconicity ratings for semantic categories of nouns, and the bottom panel shows these values for categories derived from adjectives, verbs, and the class of grammatical words and adverbs. Specific examples of words with high, low, and mixed iconicity across signed and spoken languages are presented in **Table 3**.

**Table 3:** Examples of meanings with high and low iconicity in signed and spoken languages.

Signed iconicity	Spoken iconicity	Meaning
High	High	Baby, elephant, balloon, mouth
High	Low	Book, shoulder, eat, skirt
Low	High	Wet, sick, full, slow, hard
Low	Low	Yellow, doll, apple, and
High	Mixed	Fly, pull, push, break


The row with ‘mixed’ spoken iconicity displays meanings with high iconicity in English, but low iconicity in Spanish.

As shown in the figure, among nouns in the signed languages, small artifacts (i.e., those that can be manipulated by the hands), body parts, and clothes are highest in iconicity, and among verbs, manual actions are especially high. These results demonstrate that in signed languages, iconicity is elevated in meanings related to the hands and other body parts, supporting the observation of Meir et al. (2013) that the body is ideally suited to represent itself and its parts (also Taub, 2001). On the other end of the scale, iconicity is extremely low in signs for colors. As noted above, the low iconicity of colors contributes to the negative correlation between iconicity and visual strength. Iconicity was also low in signs for time-related meanings and evaluative adjectives, as well as food, buildings and rooms, and terms for different kinds of people (including familial relationships and occupations).

In the spoken languages, over all the noun categories, Spanish words were consistently higher in iconicity than English words, with Spanish nouns for people being especially iconic. Iconicity was highest in nouns for vehicles in both English and Spanish. Outside of nouns, words for other properties were highest in iconicity in both spoken languages. Iconicity in English was also high for feelings and emotions, although this was not the case in Spanish. These results hone previous findings that adjectives, as a broad lexical class, tend to be more iconic in spoken languages, and they fit with cross-linguistic studies showing that ideophones tend to express sensory meanings (Dingemanse, 2012). Of verbs, manual actions and verbs of locomotion were highly iconic in English, but not in Spanish. This pattern may reflect a further refinement of the typological preference of English verbs to express information about manner of motion, which may be more easily rendered into iconic word forms.

Considered together, these findings suggest specific ways in which some semantic categories are more iconic in signed languages, while others are more iconic in spoken languages. Thus, they illustrate the important role of modality in determining how iconicity is distributed across the lexicon of a language. Prominently, the isomorphism between gestures and manual actions appears to motivate a heightened level of iconicity for signs mapping the two (cf. Streeck, 2009). For comparison, however, meanings related to vocal tract actions – that is, those that would afford the spoken parallel of this isomorphism (i.e., onomatopoeia) – are not represented among the 220 meanings we analyzed. Although words for sound-related meanings are generally prevalent in spoken languages, and included in our ratings for English and Spanish, vocabulary to talk about sound is presumably much less common in signed languages. Nevertheless, previous research has shown that, as a domain, sound-related words do tend to be highly iconic (Dingemanse et al., 2016; Winter et al., 2017a).

## General Discussion

Considerable evidence now shows that languages of all sorts, signed and spoken, exhibit iconicity, or resemblance between form and meaning (Perniss et al., 2010; Dingemanse et al., 2015; Perry et al., 2015). From a typological and comparative perspective (e.g., Voeltz and Kilian-Hatz, 2001; Kita and Özyürek, 2003; Dingemanse, 2012; Padden et al., 2013), this raises a host of exciting new questions regarding how iconicity is distributed across the lexicons of different languages. Some of the most basic questions to be answered relate to the modality of the language. Are signed languages really more iconic than spoken languages? How does the modality of a language influence which lexical forms are iconic and which are not? To investigate these questions, we used previously collected iconicity ratings of signs and words to compare iconicity in the vocabularies of BSL and ASL with those of English and Spanish. Our analyses produced four main sets of findings that serve to characterize how iconicity is spread across the vocabularies of the four languages. These patterns include both interesting similarities between signed and spoken languages, as well as differences between them.

First, we found positive correlations between the iconicity ratings of all four languages, including between English and both ASL and BSL, and between English and Spanish. The one notable exception to this pattern was between Spanish and both of the signed languages – perhaps reflecting the distinctly non-iconic character of Spanish verbs, which tend not to express information about the manner of movement. The relationship between the iconicity ratings of ASL and BSL was especially strong, particular in comparison to that between English and Spanish. This may indicate that the iconicity of signs is, on the whole, more direct and transparent than the iconicity of words – a point to which we return below.

Second, we found that iconicity is distributed overs signs and words in systematic ways according to an array of semantic properties. On the whole, signs and words related to the senses – meanings that are more imageable and more connected to sensory experience – are likely to be more iconic. Critically though, concreteness is only associated with more iconicity in signs, not words. Such an asymmetry makes sense, as manual gestures may provide a more concrete semiotic resource for iconicity than do vocalizations. In both types of languages, iconicity is strongest for lexical items with sensory meanings corresponding to the respective language modality – touch in signed languages, and sound in spoken languages.

Third, we found that lexical items for some semantic domains tend to be higher in iconicity than others, and there are characteristic patterns that distinguish between signed and spoken languages. These patterns of iconicity are found at the level of broad semantic categories – for example, actions, things, and properties, as reflected by English glosses as verbs, nouns, and adjectives, respectively. They are also found at the level of more specific semantic categories – manual actions, clothes, emotions, and colors, for example. For the most part, these patterns fit with predictions derived from rationale regarding the semiotic resources of sign versus speech (cf. Dingemanse et al., 2015). For example, in signed languages, signs for actions, and particularly manual actions, are quite high in iconicity, while in spoken languages, words for properties tend to be higher. Critically, this set of analyses was restricted to the 220 meanings with ratings in all four languages, and so the differences between languages cannot be attributed to differential coverage of the ratings.

Finally, one somewhat unexpected set of findings was the relatively low iconicity of nouns and visual words in signed languages, particularly those lacking connection to manual manipulation and the body. While the domain of color was an extreme case of this pattern, it does not provide the complete account. An additional explanation may be that many signs for objects may actually be limited in the level of iconicity possible, especially in comparison to the iconicity afforded by actions. For example, there may be a certain degree of abstractness involved in using the hands to represent different kinds of things, particularly those that are highly visual. This point is illustrated by the example of the ASL sign for ‘diploma’ (Taub, 2001), which combines the two hands with round handshapes that trace its rolled-up shape. Taub observed that the same iconic resources are modified to represent different kinds of cylinders – water pipes, batons, or a rolled-up poster. Although this scheme makes a productive iconic device, it also demonstrates a baseline of abstractness that derives from mapping the hands to other kinds of objects. This may drive a more moderate level of iconicity for many object meanings, even those with characteristic shapes that can be modeled with the hands.

These four sets of findings point to some interesting new directions for future research into how iconicity is distributed across different kinds of languages. However, it is important to emphasize that our conclusions are preliminary and tentative, and they should be weighed against some notable limitations of our methodology. For one, our study relied opportunistically on samples of rated signs and words that were not originally selected for cross-modal comparison. As a consequence, the ratings that overlapped across languages were somewhat lacking in systematic coverage of the semantic domains that might be of most interest. Additionally, the sample of four languages was not especially well suited to cross-modal comparison. English and Spanish – both Indo-European languages with heavy Latinate influence – are hardly representative of spoken languages. Likewise, ASL and BSL – two widely used, urban signed languages – do not represent the diversity of signed languages (de Vos and Pfau, 2015). Another notable limitation of the study is the disproportionate influence of English on our data. The norms for semantic variables were based on English glosses, rated by English speakers, and similarly, the designations of lexical class were based on English. The iconicity ratings for ASL, and many of those for BSL, were provided by non-signing English speakers. Moreover, the iconicity ratings for Spanish were provided by native speakers of Spanish who were also likely bilingual English speakers, as they were residents of the United States. Given these different factors, it is likely that more diversity in patterns of iconicity would be found by taking a more English-independent approach to a more diverse sample of signed and spoken languages.

### Comparing Iconicity in Signed and Spoken Languages

Our findings provide a preliminary, quantified account of how iconicity is spread across the lexicons of signed languages in comparison to spoken languages. As we have sought to demonstrate, the use of iconicity ratings provides researchers with a systematic, standardized method to describe how iconicity is distributed across the vocabulary of a language, which enables direct comparisons between different kinds of languages.

Notably, this approach adopts a strong theoretical premise about the nature of signs and words. The premise is not just that many signs and words are iconic, but as Wescott (1971) observed of what he called “iconism,” pointing to a quotation from Bronowski (1967, 377): “[T]he only realistic question we can ask about a given form is not ‘Is it iconic?’ but rather ‘How iconic is it?’ A measure of support for this theoretical approach to iconicity is borne out by the richness of the current results. The present work – in addition to several previous studies using iconicity ratings (e.g., Thompson et al., 2012; Perry et al., 2015; Caselli and Pyers, 2017; Occhino et al., 2017) – shows that it is useful to think of iconicity as a “substance” that can shape the forms of signs and words to a greater-or-lesser degree (Dingemanse, 2017b).

In particular, where our study breaks new ground is in its direct side-by-side comparison between signed and spoken languages. We suggest that part of the reason for the previous lack of detailed comparative studies between modalities is the widespread assumption that signed languages are far more iconic than spoken languages (Meier, 2002; Armstrong and Wilcox, 2007). For example, based largely on their intuition, Klima and Bellugi (1979, p. 21) asserted that the “vocabulary of ASL—and, to our knowledge, that of other primary sign languages—is a great deal more iconic than are the morphemes of spoken languages.” This idea figures prominently in many theories of language evolution that argue that the first symbolic forms must have been built from gestures (e.g., Corballis, 2003; Armstrong and Wilcox, 2007; Tomasello, 2008; Arbib, 2012; Fay et al., 2014). These gesture-first theories depend critically on the premise that signs afford much more iconicity than words.

Such an important claim begs for empirical evidence, and indeed, the high correlation we found here between iconicity ratings of ASL and BSL compared to English and Spanish gives it some initial quantitative support. These results suggest that iconic mappings are more consistently realized in the signs of ASL and BSL compared to the words of English and Spanish. To the extent that one can generalize from these four languages, this may indicate that signed languages are iconic in a qualitatively different – and, specifically, a more widely intuitive way – than spoken languages.

However, this intuitiveness may be limited to a significant extent. Previous research has shown that the iconic mappings of signs are not readily obvious to most naïve viewers. For example, experiments have found that non-signers are quite poor at guessing the meanings of unfamiliar signs (Klima and Bellugi, 1979). Of 90 concrete and abstract nouns from ASL, non-signers could not correctly guess the meaning of 81 of them, with the remaining signs only guessed correctly by a small proportion of participants. Guessing was only a little better when constrained to a forced choice with just five alternatives. Similarly, a more recent study of ASL with a much larger set of signs also found that non-signers were very limited in their ability to guess the meaning of the great majority of them (Emmorey and Sevcikova Sehyr, 2018). This lack of transparency is also evidenced in differences in the judgments of iconicity between signers of different languages: signers rate the signs of their native language as more iconic than those of an unfamiliar signed language (Occhino et al., 2017).

Nevertheless, despite this degree of opacity, in the experiments by Klima and Bellugi (1979), when participants were provided with the meaning of the sign, they were often very consistent in explaining the specific correspondence involved. In line with this, we found with our multiple sets of iconicity ratings for BSL that the ratings provided by non-signers were highly correlated with those provided by signers (see Materials and Methods), as was the case for ASL (Sevcikova Sehyr et al., 2017) – all with coefficients of *r* = 0.82 or higher. In comparison, Perry et al. (2015) found in two experiments with English a correlation of *r* = 0.62 between the ratings, and in two of experiments with Spanish a correlation of *r* = 0.41. While these experiments were each slightly modified in their procedure, they were alike in using only proficient speakers of the respective languages. This greater consistency in the iconicity ratings of signs may reflect the quality – perhaps reflected in a measure of concreteness – that gives traction to accounts of signed languages that postulate clearly identifiable mappings between distinct formal parameters (e.g., handshape, movement, location) and particular aspects of meaning (e.g., shape, motion, position). Such a semiotic framework has given rise to useful theoretical constructs such as structure mapping (Emmorey, 2016) and the double mapping constraint on metaphoric signs (Taub, 2001; Meir, 2010).

Yet, while iconic mappings may be more concrete and structured in signs, spoken languages do still feature their share of transparent mappings. These are found, for example, in correspondences between vowel position and size, between the vocal tract and related anatomical meanings, and between reduplication and iterative action, among others. Clearly structured mappings are especially apparent in the case of onomatopoeia, where there is potential for more isomorphic correspondence between the sound segments of a word and the properties of the sound to which it refers (e.g., Rhodes, 1994).

In addition to highly structured mappings, as our methodology highlights, signs and words may also reflect more abstract and impressionistic correspondences between form and meaning – a vaguer sense that a form looks or sounds like what it means. Thus, it may be that the iconic mappings of signed vocabularies are, on the whole, more concrete and structured, while those of spoken vocabularies are more abstract and impressionistic. Future research – using more nuanced semantic analysis to compare the iconicity of more strategically constructed samples of languages and coverage of vocabulary items – should examine this hypothesis, along with the general claim that signed languages exhibit higher overall levels of iconicity.

## Conclusion

Iconicity is now widely documented across the diverse languages of the world, signed and spoken. In both modalities, it is implicated in how people process, produce, and learn to use language, and in the evolutionary processes by which languages are created and change over time. Even if signed languages prove to be “more” iconic than spoken languages, it is becoming clear that this sort of broad generalization is no longer sufficient. Rather, we should aim to describe and compare the detailed, characteristic ways that iconicity is distributed across both kinds of languages.

Although the current study has focused on signs and words, the influence of iconicity extends far beyond the level of the lexicon. Iconicity is also pervasive in the grammars of signed and spoken languages (e.g., Givón, 1985; Liddell, 2002; Aronoff et al., 2005), and in the prosodic inflections (e.g., Shintel et al., 2006; Perlman et al., 2015; Tzeng et al., 2017) and the spontaneous bodily, oral, and vocal gestures that are deeply intertwined with signing and speaking (e.g., McNeill, 1992; Emmorey, 1999; Sandler, 2009; Kendon, 2014; Blackwell et al., 2015; Clark, 2016). Thus, a complete theory of iconicity must seek to explain how the modality of a language figures into the complex interplay between iconicity and the lexicon, as well as all of the other various levels and forms of expression that people use to communicate meaning. Only through such a comprehensive theory of iconicity will we be able to fully understand the nature of human language.

## Author Contributions

MP primarily conducted the analyses and drafted the manuscript. HL contributed to analyses and helped to revise the manuscript. BT contributed to analysis and helped to draft and revise the manuscript. RLT advised to analyses and helped to revise the manuscript.

## Conflict of Interest Statement

The authors declare that the research was conducted in the absence of any commercial or financial relationships that could be construed as a potential conflict of interest.

## Footnotes

1 We take the convention of referring to lexical items in signed languages as *signs*, and to lexical items in spoken languages as *words*.

2 Citing Klima and Bellugi (1979), Newmeyer also claimed that, “We now know that even the signed languages of the Deaf, where one might intuitively expect it, manifest little sign-referent iconicity” (p. 758). This characterization of Klima and Bellugi’s study of iconicity in ASL appears to miss the considerable complexity and nuance in the findings of their research and their conclusions.

3 www.writtensound.com

4 http://www.asl-lex.org/
